# Reply to comment on “Comparison of automatic versus constant CPAP in elderly patients after major abdominal surgery: a randomized noninferiority trial”

**DOI:** 10.1016/j.bjane.2026.844748

**Published:** 2026-03-26

**Authors:** Nguyen Dang Thu, Nguyen Thi Thuy, Le Sau Nguyen, Nguyen Ngoc Thach, Nguyen Trung Kien

**Affiliations:** a Vietnam Military Medical University, Military Hospital 103, Department of Anesthesiology, Hanoi, Vietnam; b Friendship Hospital, Department of Anesthesiology, Hanoi, Vietnam; c Vietnam Military Medical Department, Hanoi, Vietnam

*Dear Editor,*

We thank Sethuraman et al.^1^ for their thoughtful comments and the opportunity to further clarify the noninferiority framework of our study.^2^

This trial was prospectively designed to test the noninferiority hypothesis that automatic CPAP is noninferior to constant CPAP in maintaining postoperative arterial oxygenation (PaO_2_) in elderly patients after major abdominal surgery. The primary endpoint for this comparison was PaO_2_ measured during the predefined early postoperative observation period. The primary aim was therefore not to demonstrate superiority of automatic CPAP over constant CPAP, but rather to determine whether automatic CPAP could maintain postoperative oxygenation within a clinically acceptable margin compared with the standard constant CPAP mode. This noninferiority framework reflects routine clinical practice, in which both CPAP strategies are already well established as superior to conventional low-flow oxygen therapy in preventing early postoperative hypoxemia and respiratory deterioration in elderly patients undergoing major abdominal surgery. Accordingly, the clinically relevant question is whether automatic CPAP can preserve these recognized benefits without a clinically meaningful loss of efficacy, while potentially offering additional advantages in patient comfort and adaptability.

We acknowledge that a formal noninferiority margin was not explicitly prespecified in the published manuscript or in the trial registry, and we recognize this as a limitation in reporting transparency. Although the study was powered based on a clinically meaningful improvement in PaO_2_ (approximately 15 mmHg) reported in prior studies comparing CPAP or noninvasive ventilation with conventional oxygen therapy,^3^, ^4^, ^5^ this value should be understood as an assumption used for sample size estimation, rather than as a prespecified noninferiority margin for comparison between automatic and constant CPAP. In retrospect, a clearer distinction between these concepts would have better aligned the report with CONSORT recommendations for noninferiority trials.

Accordingly, the approximately 15 mmHg improvement in PaO_2_ associated with CPAP compared with conventional oxygen therapy should not be interpreted as a formal noninferiority margin, but rather as a clinically meaningful reference benchmark for the expected magnitude of benefit of CPAP-based respiratory support. In our trial, mean PaO_2_ values were 90.8 ± 12.3 mmHg with automatic CPAP and 97.5 ± 10.8 mmHg with constant CPAP, corresponding to a mean between-group difference of -6.7 mmHg (95% Confidence Interval [95% CI], -12.5 to -0.9 mmHg). When interpreted in relation to this clinically meaningful reference, the observed difference remains well within a range suggesting no clinically meaningful loss of CPAP efficacy, despite a small numerical difference between modes.

We further note that statistically significant differences observed at individual time points in repeated-measures analyses should not be interpreted as evidence of superiority in the absence of a prespecified superiority hypothesis. Such analyses primarily describe temporal patterns and within-group changes, and between-group p-values at specific time points indicate statistical differences in trajectories rather than clinically meaningful superiority. In noninferiority trials, conclusions should be guided mainly by the confidence interval of the between-group difference in relation to the noninferiority margin and by clinical relevance, rather than by isolated p-values. Because superiority testing was not prespecified in the protocol, it was therefore not formally undertaken.

Regarding graphical presentation, we agree that figures displaying confidence intervals in relation to a noninferiority margin can facilitate interpretation. However, CONSORT Item 17a notes that such figures “may be useful” rather than mandatory.^6^ The absence of such a figure therefore does not invalidate the findings, although clearer visualization might have improved interpretability for readers.

Finally, we acknowledge the concern raised in the Letter to the Editor that the absence of an explicitly stated noninferiority margin in the registry represents an important reporting issue. Prospectively, the trial was planned with a noninferiority objective and a predefined primary outcome, but the margin itself was not explicitly reported or registered. We recognize this as a limitation and appreciate the opportunity to clarify this distinction transparently.

In summary, although aspects of noninferiority reporting required clarification to meet contemporary reporting expectations, these issues pertain to transparency of presentation rather than to the conduct or validity of the study. Within a clinical context in which both CPAP strategies are known to outperform conventional oxygen therapy, our findings indicate that automatic CPAP preserves postoperative respiratory function within clinically acceptable limits and represents a reasonable alternative to constant CPAP in selected elderly patients after major abdominal surgery.

## Data availability statement

No new data were created or analyzed in this study. Data sharing is not applicable to this article.

## Authors' contributions

NDT: Conceptualization, drafting, reviewing, and editing the manuscript. NTT, LSN, NNT, NTK: Drafting the manuscript. All authors approved the final version.

## Conflicts of interest

The authors declare no conflicts of interest.
